# Molecular Prevalence and Antimicrobial Resistance Patterns of Methicillin‐Resistant *Staphylococcus aureus* in Buffaloes from Bangladesh

**DOI:** 10.1002/vms3.71295

**Published:** 2026-10-16

**Authors:** Piash Kumer Ghosh, Masud Ali, Hemayet Hossain, Md. Monjur‐e‐Alahi Tuha, Milton Roy, Tufayel Ahmed, Asikur Rahman, Md. Shahidur Rahman Chowdhury, Md. Masudur Rahman, Md. Bashir Uddin, Md. Mahfujur Rahman, Md. Mukter Hossain

**Affiliations:** ^1^ Department of Medicine Sylhet Agricultural University Sylhet Bangladesh; ^2^ Department of Veterinary Science and Animal Husbandry Teesta University Rangpur Bangladesh; ^3^ Department of Pathology Sylhet Agricultural University Sylhet Bangladesh

**Keywords:** antimicrobial resistance, Bangladesh, buffalo, methicillin‐resistant *Staphylococcus aureus*, multidrug resistance

## Abstract

**Background:**

Methicillin‐resistant *Staphylococcus aureus* (MRSA) is an emerging zoonotic pathogen in livestock and poses a potential public health threat.

**Objective:**

This cross‐sectional study investigated the prevalence, antimicrobial resistance profiles and molecular characteristics of *S. aureus* and MRSA isolated from buffalo nasal swabs in the Sylhet and Noakhali districts of Bangladesh.

**Methods:**

A total of 400 nasal swabs were collected and screened using selective culture and biochemical tests, followed by PCR targeting the *nuc* gene for *S. aureus* and the *mecA* gene for MRSA. Antimicrobial susceptibility testing (AST) was conducted using disk diffusion as per the Clinical and Laboratory Standards Institute (CLSI) 2020 standard.

**Results:**

Overall, 78 isolates (19.5%; 95% CI: 15.7–23.7) were confirmed as *S. aureus*, of which 43 (10.5%; 95% CI: 7.7–13.9) were identified as MRSA. Higher MRSA prevalence was observed in buffaloes aged 1–3 years (14.1%), males (14.8%; *p* = 0.01) and animals with respiratory symptoms (25.2%) compared to healthy buffaloes (4.3%; *p* < 0.001). AST of MRSA isolates showed high resistance to cefoxitin (97.7%), ceftriaxone (86.0%) and tetracycline (65.1%), whereas resistance to ciprofloxacin (4.7%) and levofloxacin (9.3%) was low. Multidrug resistance was detected in 88.4% (95% CI: 74.9–96.1) of MRSA isolates, with multiple antibiotic resistance index (MARI) values ranging from 0.23 to 0.54. Virulence genes (*sea*, *hla* and *icaA*) and resistance genes (*aac(3)‐IV*, *sul1*, *tetA* and *strA*) were detected.

**Conclusion:**

These findings indicate a substantial MRSA burden in buffaloes, emphasizing the need for prudent antibiotic use and ongoing surveillance to reduce zoonotic transmission risks.

## Graphical abstract

Nasal swabs collected from 400 buffaloes were analysed to determine the prevalence, antimicrobial resistance and virulence characteristics of *Staphylococcus aureus* and MRSA. MRSA isolates exhibited extensive multidrug resistance and harboured multiple resistance and virulence genes, highlighting their pathogenic and zoonotic potential. These findings emphasize the need for continuous AMR surveillance, prudent antimicrobial use, strengthened biosecurity and a One Health approach in buffalo production systems.

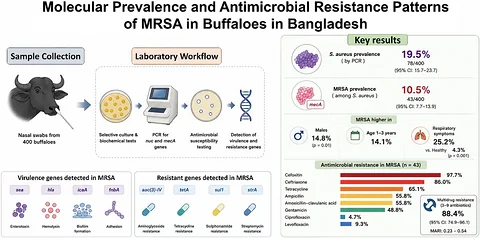

## 1 Introduction

*Staphylococcus aureus* is a major pathogen of global public health and veterinary importance, capable of colonizing multiple anatomical sites and causing diverse clinical outcomes in livestock (Chowdhury et al. 2025a). Among its various disease manifestations, methicillin‐resistant *S. aureus* (MRSA) has emerged as a particularly critical threat due to its multidrug‐resistant (MDR) phenotype, ability to persist in the farm environment and potential to spread across animal species and humans (Kumar et al. 2017). In livestock, the anterior nares and upper respiratory tract serve as key ecological niches for MRSA colonization, facilitating persistent carriage and enabling dissemination through aerosols, direct contact and contaminated fomites (Badua et al. 2020). Although MRSA has been extensively studied in cattle, especially in relation to bovine mastitis, its occurrence and epidemiological role in buffaloes remain poorly understood (Farabi et al. 2024; Chowdhury et al. 2025b). Buffaloes constitute an essential dairy and draft power species in many Asian countries, including Bangladesh, yet investigations addressing MRSA carriage in their respiratory system are virtually absent (Chowdhury et al. 2024, 2025b). The respiratory tract is a critical reservoir because colonized animals may act as silent carriers, seeding infections to other tissues or transmitting resistant strains within herds (Rasol and Abdulrahman 2023).

Importantly, *S. aureus* is one of the primary etiological agents of mastitis in dairy animals, and studies have shown that nasal carriage often precedes intramammary infection (Badua et al. 2020). Therefore, MRSA colonization of the nasal passages in buffaloes presents a plausible route for auto‐infection of the mammary gland or cross‐infection within the herd, potentially contributing to difficult‐to‐treat mastitis cases. In settings where antimicrobial use is widespread and often unregulated, respiratory carriage of MRSA also poses a zoonotic risk to farm workers and the broader community (Hoque et al. 2023).

MRSA is capable of causing a wide range of diseases, from mild skin infections to severe systemic illnesses and has been increasingly reported in both healthcare and agricultural settings (Morehead and Scarbrough 2018; Rasmi et al. 2022). Although MRSA has been well documented in humans and cattle and even isolated from mastitis cases in dairy animals, limited attention has been paid to its presence in buffaloes, especially in relation to respiratory tract infections. Several studies have confirmed the presence of MDR bacteria such as Escherichia *coli*, *S. aureus* and *Klebsiella* in livestock in Bangladesh (Bantawa et al. 2019; Liza et al. 2024; Naser et al. 2024), but these investigations have largely excluded buffaloes and have not characterized the molecular profile or resistance patterns of MRSA strains from respiratory samples.

To date, no published study in Bangladesh has systematically examined the occurrence, molecular confirmation and antimicrobial resistance (AMR) patterns of MRSA isolates from the nasal swabs of buffaloes. This represents a critical gap in the understanding of AMR epidemiology in one of the country's most valuable yet neglected livestock species. To address this gap, the present study was undertaken to isolate and identify MRSA from nasal swabs of buffaloes, to confirm MRSA at the molecular level using PCR and to assess the AMR patterns of these isolates against commonly used antibiotics. The findings of this study are intended to inform clinical management and antimicrobial stewardship strategies in buffalo health, as well as contribute to the broader understanding of zoonotic AMR risks in Bangladesh.

## 2 Materials and Methods

### 2.1 Study Area and Sample Collection

The study was conducted from April 2023 to September 2024, involving the collection of 400 nasal swab samples from buffaloes of various ages, sexes and health conditions in six upazilas (sub‐districts) across Sylhet (Zakiganj, Gowainghat, Golapganj, Jaintapur and Fenchuganj) and Noakhali (Swarna Dweep) districts of Bangladesh (Figure 1). Samples were collected randomly using sterile cotton buds directly from the nostrils and immediately placed into 50 mL containers pre‐enriched with 5 mL of Buffered Peptone Water (BPW). Samples were transported immediately to the laboratory in an icebox maintained at approximately 4°C. The individual buffalo was considered the sampling unit, and buffaloes were randomly selected from the available animals at each selected sampling site.

**FIGURE 1:**
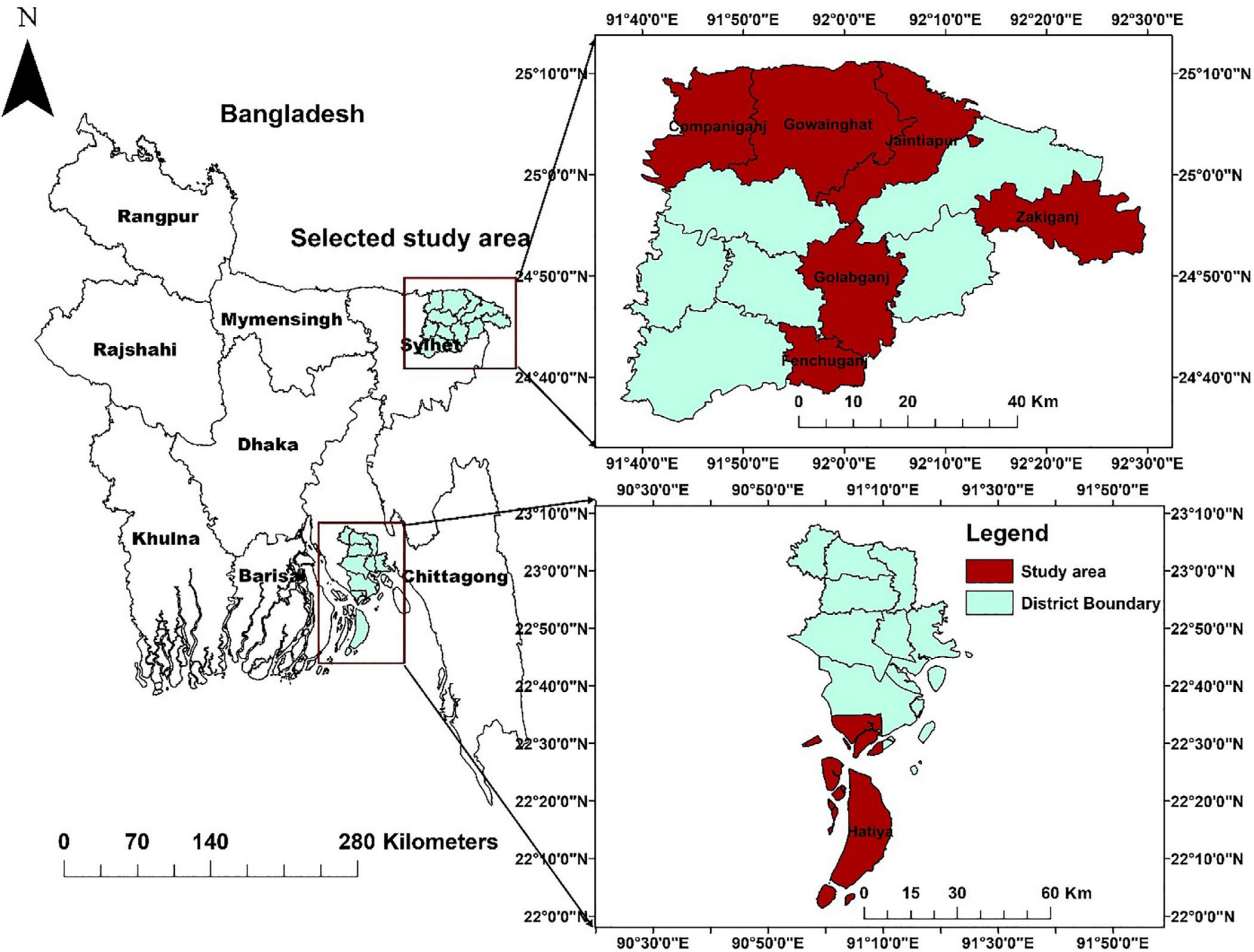
Study area mapping visualized the selected districts and sub‐districts. The map was created by ArcMap 10.8.

#### 2.1.1 Sample Size Estimation

The minimum required sample size was calculated using the following equation:

$$n=\frac{Z^{2}\times P_{\exp}\times \left(1-P_{\exp}\right)}{d^{2}}$$

where *n* is the number of required samples; *Z* = 1.96; *P*_exp_ = 0.465 (reference prevalence based on Chowdhury et al. (2025b)); *d* = 0.05, desired absolute precision (5%).

The minimum required sample size, estimated using the formula, was 382; however, this study was conducted with 400 samples using random sampling approach.

### 2.2 Bacterial Isolation and Identification

#### 2.2.1 Cultural Examination

Samples in BPW broth were incubated at 37°C for 24 h. A loopful was then streaked onto Mannitol Salt Agar (MSA) and incubated at 37°C for 18–24 h. Yellow colonies, indicative of *S. aureus*, were sub‐cultured on fresh MSA plates and subsequently on Nutrient Agar to obtain pure, opaque, mucoid, white colonies.

#### 2.2.2 Biochemical Tests

Pure isolates suspected to be *S. aureus* underwent a series of biochemical tests, including Triple Sugar Iron (TSI) agar, Methyl Red (MR) test, Simmons' Citrate Agar utilization test and Motility‐Indole‐Urease (MIU) tests, following Chowdhury et al. (2025a). Catalase testing was also performed to presumptively identify *Staphylococcus* species on the basis of their characteristic catalase activity, whereas the coagulase test was conducted to differentiate *S. aureus* from other staphylococci through the detection of coagulase production, following Farabi et al. (2024).

### 2.3 Genomic DNA Extraction

The bacterial DNA extraction was performed following the instructions provided by the manufacturer for the AddPrep Bacterial Genomic DNA Extraction Kit (ADDBIO, Korea). A 1.5‐mL micro‐centrifuge tube was filled with 1000 µL of the harvested overnight cultured sample, followed by centrifugation at 13,000 rpm for 30 s. The supernatant was then discarded. To resuspend the cell pellet, 200 µL of lysis solution and 20 µL of proteinase K (20 mg/mL) were added, either by pipetting or vortexing. The mixture was incubated at 56°C in a water bath for 10 min, with occasional vortexing to ensure thorough mixing. Subsequently, 200 µL of binding solution and 200 µL of 100% ethanol were introduced, and the mixture was vigorously vortexed for 15 s. The sample was then centrifuged at 13,000 rpm for 3 min. The supernatant, free of any pellet, was carefully transferred (600 µL) to the upper reservoir of a spin column. The spin column was placed in a collection tube, and 500 µL of washing solution 1 was added before centrifugation at 13,000 rpm for 1 min. The flow‐through was discarded, and the spin column was transferred to a 2.0‐mL collection tube. Next, 500 µL of Washing Solution 2 was added, followed by another 1‐min centrifugation at 13,000 rpm. After discarding the flow‐through, the spin column was again placed in the 2.0 mL collection tube. To remove any residual ethanol, a final centrifugation step was performed at 13,000 rpm for 1 min. The spin column was then transferred to a fresh 1.5 mL micro‐centrifuge tube. To elute the genomic DNA, 50 µL of elution solution was added to the spin column and allowed to sit for at least 1 min. The DNA was subsequently eluted by centrifugation at 13,000 rpm for 1 min. The purified DNA was stored at −20°C for immediate use.

### 2.4 PCR Amplification for *S. aureus* and MRSA Confirmation

PCR was performed to confirm *S. aureus* by amplifying the *nuc* gene (359 bp and to detect MRSA by amplifying the *mecA* gene (286 bp product). Primer sequences and references are provided in Table 1.

**TABLE 1:** The primer sequences, amplicon sizes and target genes for the detection of *Staphylococcus aureus*, antimicrobial resistant genes (ARGs) and virulent genes of isolated *S. aureus* from nasal swabs of buffaloes.

Genotype category	Target genes	Primer sequence (5′–3′)	Amplicon size (bp)	Annealing temp. (°C)	References
*Staphylococcus aureus*	*nuc*	F: GCGATTGATGGTGATACGGTT	359	56	Mehrotra et al. (2000)
		R: AGCCAAGCCTTGACGAACTAAAGC			
MRSA	*mecA*	F: TGCTATCCACCCTCAAACAGG	286	55	Kondo et al. (2007)
		R: AACGTTGTAACCACCCCAAGA			
ARGs	*aac(3)‐IV*	F: AGTTGACCCAGGGCTGTCGC	333	63	Heuer et al. (2002)
		R: GTGTGCTGCTGGTCCACAGC			
	*tetA*	F: GCTACATCCTGCTTGCCTTC	502	63	Karczmarczyk et al. (2011)
		R: CATAGATCCCGTGAAGAGG			
	*strA*	F: ATGGTGGACCCTAAAACTCT	893	63	Kozak et al. (2009)
		R: CGTCTAGGATCGAGACAAAG			
	*sul1*	F: CGGCATCGTCAACATAACCT	433	66	Lanz et al. (2003)
		R: TGTGCGGATGAAGTCAGCTC			
Virulent genes	Haemolysin A (*hla*)	F: CTGATTACTATCCAAGAAATTCGATTG	209	58	Rasmi et al. (2022)
		R: CTTTCCAGCCTACTTTTTTATCAGT			
	Staphylococcal enterotoxin A (*sea*)	F: TTGGAAACGGTTAAAACGAA	120	55	
		R: GAACCTTCCCATCAAAAACA			
	Intracellular adhesion‐A (*ica*A)	F: GATTATGTAATGTGCTTGGA	770	50	
		R: ACTACTGCTGCGTTAATAAT			
	Fibronectin‐binding protein‐A (*fnb*A)	F: GCGGAGATCAAAGACAA	1279	47	
		R: CCA TCT ATA GCT GTG TGG			

*Abbreviation*: MRSA, methicillin‐resistant *Staphylococcus aureus*.

PCR amplification was performed in a total reaction volume of 25 µL, consisting of 10 µL of master mix, 10 µL of DNA template and 5 µL of the primer working solution. The reaction mixture was prepared on ice and mixed thoroughly before thermal cycling.

For amplification of the *nuc (S. aureus)* gene, PCR was initiated with an initial denaturation at 95°C for 5 min, followed by 30 cycles of denaturation at 95°C for 30 s, annealing at 56°C for 30 s and extension at 72°C for 15 s, with a final extension at 72°C for 5 min before holding at 4°C. For amplification of the *mecA* (MRSA) gene, PCR was performed with an initial denaturation at 94°C for 5 min, followed by 30 cycles of denaturation at 94°C for 1 min, annealing at 55°C for 45 s and extension at 72°C for 45 s, followed by a final extension at 72°C for 5 min and a final hold at 4°C. The amplified PCR products were resolved on 1.5% agarose gel, visualized under UV transillumination and documented using a gel documentation system. A confirmed positive isolate from the laboratory repository was used as the positive control, whereas the negative control contained all PCR components except the DNA template, that is, PCR master mix, primers and nuclease‐free water.

### 2.5 PCR for AMR Genes

The selected AMR genes represent resistance to commonly used antibiotic classes in both veterinary and human medicine. The *aac(3)‐IV* gene confers resistance to aminoglycosides, particularly gentamicin, whereas *tetA* mediates tetracycline resistance through an efflux mechanism. The *strA* gene is associated with streptomycin resistance, whereas *sul1* confers resistance to sulphonamides and is frequently linked to class 1 integrons, facilitating the dissemination of AMR (Bhat et al. 2023).

PCR assays were conducted to detect specific AMR genes: *aac(3)‐IV* (gentamicin resistance, 350 bp), *tetA* (502 bp), *strA* (893 bp) and *sul1* (433 bp). Primer sequences and references are provided in Table 1. Each PCR reaction was carried out in a final volume of 25 µL containing 10 µL of 2× PCR Master Mix, 10 µL of template DNA and 5 µL of primer working solution. For amplification of the *aac(3)‐IV* gene, PCR was initiated with an initial denaturation at 94°C for 10 min, followed by 35 cycles of denaturation at 94°C for 1 min, annealing at 63°C for 1 min and extension at 72°C for 1 min, with a final extension at 72°C for 10 min. The *tetA* and *strA* genes were amplified using the same cycling conditions with an annealing temperature of 63°C, whereas the *sul1* gene was amplified under identical conditions except that the annealing temperature was 66°C. PCR products were separated on 1.5% agarose gel, stained, visualized under a UV transilluminator and documented using a gel documentation system.

### 2.6 PCR for Virulence Genes

The selected virulence genes represent key determinants involved in the pathogenicity of *S. aureus*. The *hla* gene encodes α‐haemolysin, a pore‐forming cytotoxin that contributes to host cell damage and tissue invasion. The *sea* gene encodes staphylococcal enterotoxin A, a heat‐stable superantigen associated with food poisoning. The *icaA* gene is involved in biofilm formation through the synthesis of polysaccharide intercellular adhesin, whereas the *fnbA* gene encodes fibronectin‐binding protein A, which mediates bacterial adhesion, colonization and invasion of host tissues (Girma 2024).

Multiplex PCR was employed to detect virulence genes: haemolysin A (*hla*, 209 bp), staphylococcal enterotoxin A (*sea*, 120 bp), intracellular adhesion‐A (*icaA*, 770 bp) and fibronectin‐binding protein‐A (*fnbA*, 1279 bp). Primer sequences and product sizes are listed in Table 1. Reaction mixtures and cycling conditions were optimized for each gene accordingly. Amplified products were visualized using 1.5% agarose gel electrophoresis and a UV transilluminator.

#### 2.6.1 PCR Cycling Condition for Virulent Gene Amplification

The haemolysin A (*hla*) gene was identified using u‐PCR, which involved a 5‐min initial denaturation at 95°C, 40 cycles of denaturation at 95°C for 50 s, 30 s annealing at 58°C and at 72°C for 1 min for elongation and a 10‐min final elongation step at 72°C. Likewise, the staphylococcal enterotoxin A (*sea*) gene was identified using u‐PCR. The protocol involved a 5‐min initial denaturation stage at 95°C, 40 cycles of denaturation at 95°C for 1 min, 45 s of annealing at 55°C and 72°C for 1 min for elongation and a 10‐min final elongation step at 72°C. Similarly, intracellular adhesion‐A (*icaA*) gene was identified using u‐PCR, that involved a 5‐min initial denaturation at 95°C, 40 cycles of denaturation at 95°C for 1 min, annealing at 50°C for 1 min, elongation at 72°C for 1.5 min and final elongation at 72°C for 5 min. Fibronectin‐binding protein‐A (*fnbA*) gene was also performed which involved a 5‐min initial denaturation at 95°C, 40 cycles of denaturation at 95°C for 1 min, annealing at 47°C for 1 min, elongation at 72°C for 1.5 min and final elongation at 72°C for 5 min.

### 2.7 Agarose Gel Electrophoresis

After making a 100‐mL 1.5% agarose solution (a blend of 1× TAE buffer and agarose LE, produced by ADD BIO INC, Korea), the liquid was chilled to between 60°C and 70°C. After that, the mixture was gently stirred (to prevent the formation of water bubbles), and the gel was cast into a gel plate with a comb inserted into one of its poles. The gel plate was set atop the gel electrophoresis tank after it had solidified so that the gel was submerged between 2 and 5 mm. The gel holes were filled with samples. The gel was tested under a UV lamp for 30 min once it was initially treated at 100 volts.

### 2.8 Antimicrobial Susceptibility Testing (AST)

The Kirby–Bauer disk diffusion method was performed on Mueller–Hinton agar to assess the antibiotic susceptibility of MRSA isolates, following Clinical and Laboratory Standards Institute (CLSI 2020) guidelines. A panel of 13 widely used antibiotics from 8 antimicrobial classes were tested. Zones of inhibition were measured and interpreted as sensitive, intermediate or resistant (Table S1). Quality control (QC) of AST was performed using *S. aureus* ATCC 25923 in accordance with CLSI guidelines to ensure the accuracy and reliability of inhibition zone measurements.

### 2.9 MDR and Multiple Antibiotic Resistance (MAR) Index Determination

MDR was defined as resistance to three or more antimicrobial classes (Magiorakos et al. 2012). The MAR index was calculated by dividing the number of antibiotics to which an isolate was resistant by the total number of antibiotics tested (Islam et al. 2025; Chowdhury et al. 2025a). The MAR index ranges from 0 to 1, where a value of 0 indicates that the isolate is sensitive to all tested antibiotics, and a value of 1 indicates resistance to all tested antibiotics. A MAR index ≤0.2 suggests that antibiotics are used in a controlled environment and that the isolate likely originates from a low‐risk source. In contrast, an MAR index >0.2 suggests that the isolate originates from a high‐risk source with frequent or uncontrolled antimicrobial use (Islam et al. 2025).

### 2.10 Plotting and Geographical Mapping

A heatmap was generated using the open‐source online platform ChiPlot (www.chiplot.online). The distribution of ARGs and virulence genes was visualized using Microsoft Excel. Geographical maps were generated with ArcMap version 10.8 (ESRI, USA). Shapefiles were extracted from the Global Administrative Areas database (GADM) (https://gadm.org/) and incorporated into ArcMap for visualization.

### 2.11 Statistical Analysis

Primary data were organized in Microsoft Excel and analysed for descriptive statistics. Categorical variables were summarized as frequencies with confidence intervals. Associations between categorical variables were assessed using the chi‐square test or Fisher's exact test, where appropriate. Exact binomial methods were used to estimate 95% confidence intervals. All the statistical analysis was performed using R 4.5.1. A *p* value <0.05 was considered statistically significant.

## 3 Results

A total of 400 nasal swab samples were collected from buffaloes, of which 78 (19.5%; 95% CI: 15.7–23.7) were confirmed as *S. aureus* by PCR targeting the *nuc* gene. Among these, 43 isolates (10.5%; 95% CI: 7.7–13.9) were identified as MRSA through the detection of the *mecA* gene (Table 2).

**TABLE 2:** Overall molecular prevalence of *Staphylococcus aureus* and methicillin‐resistant *S. aureus* (MRSA) isolated from buffalo nasal swab.

Pathogens type	*x*/*N*	%	95% CI
*Staphylococcus aureus*	78/400	19.5	15.7, 23.7
MRSA	43/400	10.5	7.7, 13.9

### 3.1 Prevalence of MRSA in Relation to Epidemiological Variables

The prevalence of MRSA varied across study locations, age groups and health status of buffaloes (Table 3). Geographically, prevalence ranged from 5.36% in Zakiganj to 14.55% in Swarna Dweep. Age‐wise analysis showed the highest prevalence (14.13%) among buffaloes aged 1–3 years, compared with 6.25% in calves (<1 year) and 8.65% in older animals (>3 years). A statistically significant difference was observed between sexes, with males (14.83%) exhibiting higher MRSA prevalence compared to females (6.88%) (*p* = 0.01). Health status was strongly associated with MRSA occurrence: Buffaloes with respiratory symptoms showed a significantly higher prevalence (25.21%) compared to clinically healthy animals (4.27%) (*p* < 0.001).

**TABLE 3:** Prevalence of methicillin‐resistant *Staphylococcus aureus* (MRSA) isolated from nasal swabs of buffaloes in Bangladesh.

Variable name	Total sample tested	Positive samples	Prevalence (%)	95% CI	*χ* ^2^ value	*p* value
Study area						
Zakiganj	56	3	5.36	1.1, 14.9	4.53	0.61
Gowainghat	52	7	13.46	5.6, 25.8		
Jaintapur	66	5	7.58	2.5, 16.8		
Golapganj	53	6	11.32	4.3, 23.0		
Fenchuganj	68	9	13.23	6.2, 23.6		
Companiganj	50	4	8.00	2.2, 19.2		
Swarna Dweep	55	8	14.55	6.5, 26.7		
Age						
<1 year	112	7	6.25	2.6, 12.5	5.11	0.078
1–3 years	184	26	14.13	9.4, 20.0		
>3 years	104	9	8.65	8.0, 9.6		
Sex						
Male	182	27	14.83	14.3, 15.5	6.68	0.01
Female	218	15	6.88	3.9, 11.1		
Health status						
Buffalo with respiratory symptoms	119	30	25.21	17.7, 33.9	39.0	<0.001
Healthy buffaloes	281	12	4.27	2.2, 7.3		

### 3.2 Antimicrobial Susceptibility Patterns of MRSA

Phenotypic AST of MRSA isolates (*n* = 43) revealed high levels of resistance to several commonly used antibiotics (Table 4). Resistance was most prominent to cefoxitin (97.7%) and ceftriaxone (86.0%) following tetracycline (65.12%) and ampicillin (58.14%). Moderate resistance was observed against trimethoprim‐sulphamethoxazole (41.86%) and gentamicin (48.84%). In contrast, lower resistance rates were detected for amikacin (9.30%), azithromycin (9.30%), ciprofloxacin (4.65%) and levofloxacin (9.30%). Notably, a majority of isolates remained sensitive to fluoroquinolones, with 81.40% sensitivity to ciprofloxacin and 86.05% to levofloxacin. The heatmap depicted the highly resistant group of antibiotics (cefoxitin, ceftriaxone and tetracycline) in the same cluster and the sensitive group (ciprofloxacin, levofloxacin, azithromycin, etc.) in another cluster (Figure 2A).

**TABLE 4:** Results of phenotypic antibiotic susceptibility test of methicillin‐resistant *Staphylococcus aureus* (*N* = 43) isolated from nasal swabs of buffaloes.

Antimicrobial class	Antibiotics	Interpretation		
		Sensitive (%)	Intermediate (%)	Resistant (%)
Penicillin	Ampicillin (AMP)	32.56	9.30	58.14
	Amoxicillin/Clavulanic acid (AMC)	32.56	11.63	55.81
Cephems, including cephalosporin	Cefoxitin (FOX)	0	2.30	97.70
	Ceftriaxone (CRO)	4.70	9.30	86.0
Aminoglycosides	Gentamicin (CN)	41.86	9.30	48.84
	Amikacin (AK)	79.07	11.63	9.30
	Streptomycin (STR)	65.12	20.93	13.95
Tetracyclines	Tetracycline (TET)	30.23	4.65	65.12
Macrolides	Azithromycin (AZM)	74.42	16.28	9.30
Quinolones	Ciprofloxacin (CIP)	81.40	13.95	4.65
	Levofloxacin (LEV)	86.05	4.65	9.30
Folate pathway antagonists	Trimethoprim‐sulphamethoxazole (SXT)	18.60	39.53	41.86
Phenicol	Chloramphenicol (C)	60.47	20.93	18.60

**FIGURE 2:**
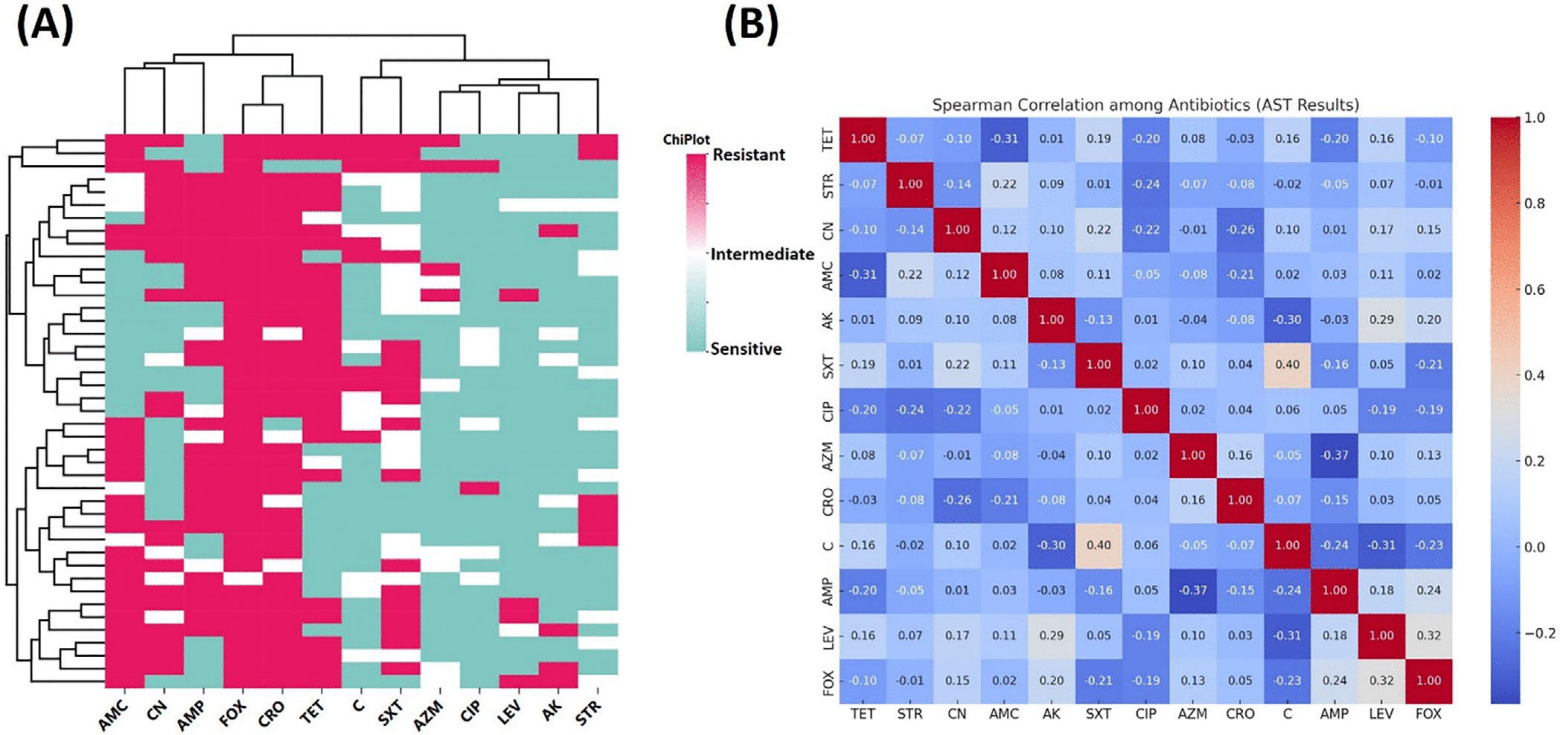
Antimicrobial resistance pattern of the isolated MRSA (*N* = 43). Heatmap showing the distribution pattern of the isolates (A). The Spearman correlation among the tested antibiotics (B). AST, antimicrobial susceptibility testing.

The Spearman correlation analysis revealed important associations among the resistance patterns of different antibiotics (Figure 2B). Strong positive correlations indicate that resistance to one antibiotic is often accompanied by resistance to another, possibly due to cross‐resistance or shared resistance mechanisms, whereas weak or negative correlations suggest independent resistance profiles.

### 3.3 Phenotypic Resistance Patterns and MAR Index

Out of the 43 MRSA isolates, 38 (88.4%; 95% CI: 74.9–96.1) were classified as MDR‐(MRSA), exhibiting resistance to three or more classes of antibiotics (Table 5). The multiple antibiotic resistance index (MARI) of 38 MDR MRSA ranged from 0.23 to 0.54, indicating exposure to high‐risk environments with frequent antibiotic use.

**TABLE 5:** Descriptive comparison of phenotypic resistance patterns, detected antimicrobial resistance genes (ARGs), resistance type and multiple antibiotic resistance (MAR) index among multidrug‐resistant (MDR)‐methicillin‐resistant *Staphylococcus aureus* (MRSA) isolates (*N* = 38).

Organisms	No. of isolates	Phenotypic pattern	Antibiotics (class)	Resistance type	ARGs	MARI
**MRSA**	1	TET‐CN‐AMP‐FOX	4 (4)	MDR	*tetA, aac‐3(iv)*	0.31
	1	CN‐AMP‐SXT‐CRO‐FOX‐C	6 (5)	MDR	*aac‐3(iv)*	0.46
	3	TET‐AMC‐AMP‐SXT‐LEV‐CRO‐FOX	7 (5)	MDR	*tetA, aac‐3(iv), sul1*	0.54
	1	STR‐AMC‐AMP‐FOX	4 (3)	MDR	*strA*	0.31
	2	TET‐AMP‐SXT	3 (3)	MDR	*sul1*	0.23
	1	TET‐CN‐AMC‐AMP‐SXT‐LEV‐FOX	7 (6)	MDR	*sul1*	0.54
	1	CN‐AMC‐AMP‐SXT	4 (3)	MDR	*aac‐3(iv)*	0.31
	1	AMP‐CIP‐CRO‐FOX	4 (3)	MDR		0.31
	1	CN‐AK‐AMC‐AMP‐SXT‐FOX	6 (4)	MDR		0.46
	2	TET‐AMC‐FOX‐C	4 (4)	MDR	*tetA*	0.31
	1	TET‐AMP‐CRO	3 (3)	MDR		0.23
	1	TET‐AK‐AMC‐LEV‐FOX	5 (5)	MDR		0.38
	1	TET‐STR‐AMC‐SXT‐C	5 (5)	MDR	*strA*	0.38
	1	TET‐SXT‐CRO‐FOX‐C	5 (4)	MDR	*sul1*	0.38
	1	TET‐SXT‐CRO‐C	4 (4)	MDR	*sul1*	0.31
	2	TET‐CN‐AMP‐FOX	4 (4)	MDR	*tetA, aac‐3(iv)*	0.31
	1	TET‐CN‐AMC	3 (3)	MDR	*aac‐3(iv)*	0.23
	1	STR‐CN‐AMC‐AMP‐FOX	5 (3)	MDR	*strA*	0.38
	1	TET‐CN‐AK‐AMC‐SXT‐CRO‐FOX	7 (5)	MDR	*aac‐3(iv)*	0.54
	1	TET‐AMP‐AZM‐FOX	4 (4)	MDR	*tetA*	0.31
	1	TET‐AMC‐AMP‐SXT‐FOX	5 (4)	MDR	*tetA, sul1*	0.38
	1	CN‐AMP‐FOX	3 (3)	MDR		0.23
	1	TET‐STR‐CN‐AMC‐SXT‐AZM‐C	7 (6)	MDR	*aac‐3(iv), strA*	0.54
	1	TET‐CN‐AMC‐SXT	4 (4)	MDR	*tetA*	0.31
	1	CN‐AMC‐SXT‐CIP‐AZM‐FOX‐C	7 (7)	MDR	*aac‐3(iv), sul1*	0.54
	1	TET‐AMC‐AMP‐SXT	4 (3)	MDR		0.31
	1	TET‐CN‐AMP‐FOX	4 (4)	MDR	*aac‐3(iv)*	0.31
	1	TET‐CN‐AMC‐AMP‐FOX‐C	6 (5)	MDR		0.46
	1	TET‐CN‐AK‐AMC‐AMP‐FOX	6 (5)	MDR	*aac‐3(iv)*	0.46
	1	TET‐AMP‐SXT	3 (3)	MDR	*sul1*	0.23
	1	TET‐CN‐SXT	3 (3)	MDR		0.23
	1	CN‐AMC‐SXT‐FOX	4 (3)	MDR		0.31
	1	TET‐CN‐AMP‐LEV‐AZM‐FOX	6 (6)	MDR	*aac‐3(iv)*	0.46
**Total**	38					

*Abbreviation*: AK, amikacin; AMC, amoxicillin/clavulanic acid; AMP, ampicillin; AZM, azithromycin; C, chloramphenicol; CIP, ciprofloxacin; CN, gentamicin; CRO, ceftriaxone; FOX, cefoxitin; LEV, levofloxacin; MARI, multiple antibiotic resistance index; STR, streptomycin; SXT, trimethoprim‐sulphamethoxazole; TET, tetracycline.

### 3.4 Distribution of Virulence Genes

Screening for virulence‐associated genes among *S. aureus* isolates (*n* = 78) revealed variable prevalence (Table S2). The *sea* gene (Staphylococcal enterotoxin A) was most frequently detected (17.94%), followed by *hla* (haemolysin A; 11.53%) and *icaA* (intracellular adhesion A; 6.41%), as shown in Figure 3. None of the isolates carried the *fnbA* gene (fibronectin‐binding protein A).

**FIGURE 3:**
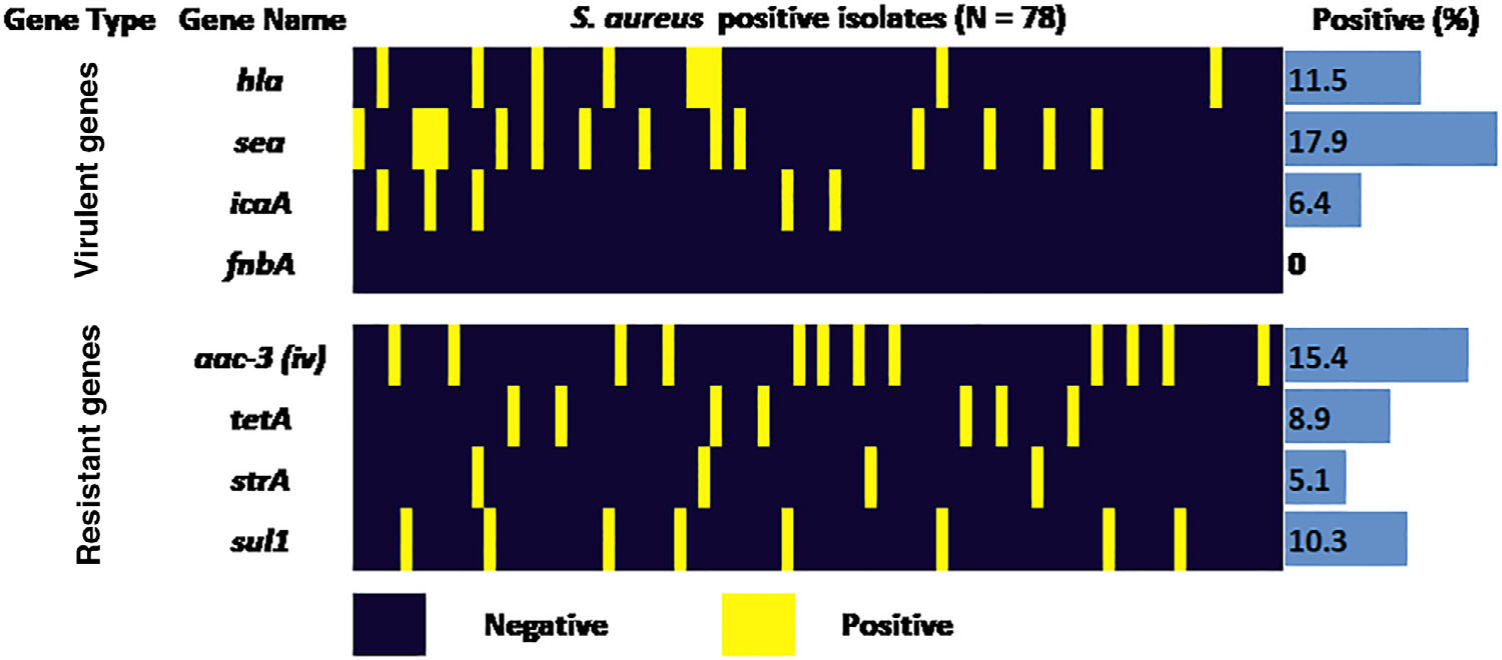
Distribution of virulent and antimicrobial resistance genes among the positive isolates of *Staphylococcus aureus* (*N* = 78).

### 3.5 Distribution of ARGs

Molecular analysis of ARGs in *S. aureus* isolates showed the highest prevalence of *aac(3)‐IV* (15.38%), followed by *sul1* (10.25%), *tetA* (8.97%) and *strA* (5.12%) (Figure 3; Table S3). Several MDR isolates harboured multiple ARGs, supporting the phenotypic resistance patterns observed.

## 4 Discussion

The findings of this study provide crucial baseline data on high prevalence, AMR and virulent gene carriage in *S. aureus* isolated from nasal swabs of buffaloes, the majority of which were found to be MRSA. In One Health approach, the findings have important implications for livestock production, human and animal health and antibiotic use. Our research adds to the expanding literature on livestock‐associated MRSA by demonstrating that buffaloes can serve as an MRSA reservoir. It also draws attention to a neglected host species where nasal swab may be a key determinant in the survival and spread of the pathogen.

Normally, MRSA spreads from dairy mastitis and humans, but the finding of MRSA‐positive samples also indicates possible spread via nasal swab. Due to the intensive farming system, buffaloes become more susceptible to spreading ARGs. Some factors, such as increased stock density, misuse of antibiotics and poor biosecurity, may be responsible for this. Our findings support past reports of MRSA in livestock, especially in pigs and cattle (Pantosti 2012), but they also apply to nasal swabs of buffalo, a relatively unstudied sample. A signific finding in this study is the overall prevalence of MRSA (10.50% of PCR‐positive *S. aureus* isolates) and *S. aureus* (19.5% by PCR) in the buffaloes (Rasmi et al. 2022).

From the study, 10.50% of isolates were MRSA which indicates a considerable public health concern. Previously another study states that MRSA prevalence in livestock ranges from 0% to 46.29% (Barua et al. 2025). One study in Egypt found that MRSA prevalence in water buffalo was close to 43.6% (Selim et al. 2025), whereas another study noted MRSA prevalence in cattle in China was 18% (Zhang et al. 2005). Availability and misuse of antibiotics, less veterinary supervision and the presence of ARGs in agricultural sector might be the reasons (Argudín et al. 2010). The findings are especially pertinent because historically the nasal swab has received less attention as a sampling niche than milk or skin.

Between Zakiganj and Swarna Dweep, the MRSA frequently varied from 5.36% to 14.55%, respectively. Although geographical variations were not highly significant, they suggest that patterns of antibiotic use, managemental practices of farms and environmental conditions may have an impact. Previous investigations on the prevalence of MRSA in bovine populations have observed similar regional heterogeneity, demonstrating the significance of targeted surveillance and intervention programmes (Hoque et al. 2022).

Buffaloes under 1 year old (6.25%) and those over 3 years old (8.65%) had the lowest MRSA prevalence, followed by those aged 1–3 years old (14.13%). The pattern indicates that higher MRSA colonization may be caused by increased environmental exposure and potential antimicrobial treatments during the early productive years (1–3 years). These results are in part consistent with previous research that demonstrated that higher levels of stress and antimicrobial exposure in adult animals can lead to the establishment of resistance strains (Liang et al. 2019). Our findings also imply that young buffaloes less than 1 year old do not have a high level of colonization which may be because of maternal immunity or a lack of exposure to environmental pathogens and antibiotics.

There was a significant correlation between MSA prevalence and sex, with male buffaloes exhibiting a positive rate of 14.83%, whereas females showed 6.88% rate. In cattle and small ruminants, similar sex‐related variations in bacterial transmission have been documented (Vanderhaeghen et al. 2010; Monecke et al. 2011). This goes somewhat against what is expected because females, particularly breeding animals, frequently receive more veterinary care and antibiotic treatments, which may encourage resistance. This male‐dominant trend, however, could be the result of behavioural, physiological or management‐related differences, such as greater environmental exposure, increased physical stress or hormonal influences on immunity (Hoque et al. 2022). This male dominant trend, however, can be the result of behavioural, physiological or management variations, such as increased outside exposure, increased physical stress or hormonal effects on immunity. The slightly higher prevalence in male buffaloes and certain age groups could be influenced by variations in exposure, physiological stress or immunological status (Beyene et al. 2017).

MRSA has been shown to have the highest correlation with animal health. The prevalence of respiratory symptoms in buffaloes was 25.21% which was substantially greater than that of healthy buffaloes 4.27%. This discovery corroborates earlier findings that MRSA is frequently linked to clinical illness, specifically respiratory and wound infections in animals (Fessler et al. 2010). According to the findings, MRSA may infect vulnerable hosts as an opportunistic colonizer or as a main respiratory infection.

Antibiotics susceptibility testing showed that MRSA isolates exhibited extensive MDR. As anticipated, *mecA‐*mediated resistance was confirmed by the high level of resistance to beta‐lactams, with 97.7% resistant to cefoxitin, followed by ceftriaxone (86.0%), and over 55% resistant to ampicillin and amoxicillin‐clavulanic acid. Notably, tetracycline resistance was remarkably high (65.1%), which is consistent with its widespread uses in veterinary medicine. Despite amoxicillin's great activity (79.1% susceptible), gentamicin resistance (48.8%) was also significant, most likely as a result of its restricted veterinary use (Chowdhury et al. 2021). The most effective antibiotics were quinolones, specially ciprofloxacin (81.4% susceptible) and levofloxacin (86.1% susceptible), indicating their potential for therapeutic use. However, the necessity for cautious use is highlighted by *S. aureus*’s tendency to quickly develop fluoroquinolone resistance (Paterson et al. 2014). These resistance patterns are consistent with studies of livestock‐associated MRSA worldwide, where MDR is now prevalent (Fessler et al. 2010).

The pathogenic potential of MRSA associated with buffalo was further validated by genomic research. In line with studies in bovine MRSA, adhesion and immune evasion genes are among the most common virulence determinants (17.9% and 11.5%, respectively), indicating a tendency for persistent colonization rather than acute toxin‐mediated illness (Vanderhaeghen et al. 2010). Although they were less common (6.4%), the existence of toxin‐associated gene suggests that a subset of isolates may be responsible for more severe disease.

The study identified serious AMR issues by analysing MRSA from nasal swabs of buffalo in Bangladesh. Reflecting extensive antibiotic exposure in livestock, among the 43 MRSA isolates, 38 (88.4%) exhibited resistance to 3–9 antibiotics, with cefoxitin, ceftriaxone, tetracycline and ampicillin being the most often resistant (Chowdhury et al. 2025b). The resistance genes *tetA, aac‐3(iv), sul1* and *strA* were shown to frequently co‐occur by molecular analysis, indicating complicated resistance mechanism through clonal propagation or horizontal gene transfer (Parvin et al. 2021). According to Islam et al. (2025), there were virulence‐associated genes, such as *bap, seb, pvl, hla* and *hlb*, which suggest possible pathogenicity and a risk of serious infections in both people and animals. However, this study investigated the following virulence genes *hla, sea, icaA* and *fnbA*. To show the spread of AMR, integrated One Health approaches are crucial, including prudent antibiotic use and surveillance (Ara et al. 2025).

This study highlights the urgent need for robust antimicrobial stewardship programmes in Bangladesh's buffalo farming sector. Promoting judicious use of antibiotics, implementing stringent biosecurity measures and enhancing surveillance for AMR are crucial steps to control the spread of resistant bacteria. The potential for cross‐contamination and transmission between animals and humans emphasizes the importance of a One Health approach to combat AMR effectively.

## 5 Limitations

This study has some limitations, including the relatively small sample size, which may have reduced statistical power for subgroup analyses. Risk‐factor analysis using ORs with 95% CIs and multivariable analysis was not performed, as it was beyond the primary scope of this study. The cross‐sectional design limits the assessment of temporal changes and transmission dynamics of MRSA. Additionally, MIC testing, advanced molecular typing (MLST and SCCmec) and *mecC* gene screening were not performed, limiting detailed characterization of resistance mechanisms and genetic diversity. The absence of antimicrobial usage data and the use of nasal swabs only may have restricted the evaluation of risk factors and overall MRSA colonization patterns. Further studies with larger populations, longitudinal designs and comprehensive molecular approaches are recommended.

## 6 Conclusions

This study reveals a considerable nasal carriage of *S. aureus* and MRSA among buffaloes in Bangladesh, with MRSA detected in 10.5% of animals. MRSA prevalence varied by location and was significantly higher in males and buffaloes with respiratory symptoms, indicating important epidemiological and health‐associated factors. The isolates showed high resistance to commonly used antibiotics, particularly β‐lactams, cephalosporins and tetracycline, while retaining greater susceptibility to fluoroquinolones and amikacin. The high proportion of MDR MRSA, elevated MAR index and presence of virulence and resistance genes underscore selective antimicrobial pressure. These findings highlight the need for routine surveillance, rational antimicrobial use and improved herd health management to mitigate MRSA potential spread.

## Author Contributions

Conceptualization: Md. Mukter Hossain, Md. Bashir Uddin and Md. Mahfujur Rahman. Methodology: Md. Mukter Hossain, Piash Kumer Ghosh, Masud Ali, Md. Masudur Rahman and Md. Mahfujur Rahman. Software: Hemayet Hossain, Md. Mukter Hossain and Md. Mahfujur Rahman. Validation: Md. Mukter Hossain, Md. Bashir Uddin, Piash Kumer Ghosh, Md. Monjur‐e‐Alahi Tuha, Masud Ali and Md. Mahfujur Rahman. Formal analysis: Md. Mukter Hossain, Hemayet Hossain, Md. Mahfujur Rahman, Milton Roy, Asikur Rahman, Tufayel Ahmed and Md. Monjur‐e‐Alahi Tuha. Investigation: Md. Mukter Hossain, Md. Bashir Uddin, Md. Shahidur Rahman Chowdhury and Md. Mahfujur Rahman. Resources: Md. Mukter Hossain and Md. Mahfujur Rahman. Data curation: Piash Kumer Ghosh, Masud Ali, Asikur Rahman, Tufayel Ahmed, Md. Monjur‐e‐Alahi Tuha, Md. Shahidur Rahman Chowdhury and Md. Mukter Hossain. Writing; original draft preparation: Md. Mukter Hossain, Piash Kumer Ghosh, Masud Ali, Hemayet Hossain, Milton Roy, Md. Shahidur Rahman Chowdhury, Md. Bashir Uddin and Md. Mahfujur Rahman. Writing, review and editing: Md. Mukter Hossain, Hemayet Hossain, Md. Mahfujur Rahman, Masud Ali, Piash Kumer Ghosh, Asikur Rahman, Tufayel Ahmed, Md. Bashir Uddin and Md. Masudur Rahman. Visualization: Md. Mukter Hossain and Md. Bashir Uddin. Supervision: Md. Mukter Hossain, Md. Mahfujur Rahman, Md. Bashir Uddin and Md. Masudur Rahman. Project administration: Md. Mukter Hossain and Md. Mahfujur Rahman. Funding acquisition: Md. Mukter Hossain and Md. Mahfujur Rahman. All authors have read and agreed to the published version of the manuscript.

## Funding

This research was funded by the Ministry of Science and Technology (MoST) of Bangladesh, grant number: SRG‐251272 as well as partially funded by the University Grant's Commission (UGC) of Bangladesh, grant number: UGC‐Lifescience‐96/2021‐22.

## Ethics Statement

The Animal Experimentation and Ethics Committee (AEEC) of Sylhet Agricultural University, Sylhet, gave its approval for this study along with the animal use protocol number (#AUP2021052). Animal handling and welfare procedures were conducted in accordance with standard animal welfare practices and reported in accordance with the relevant ARRIVE 2.0 recommendations.

## Supporting information




**Table S1**: Prevalence of virulence genes of *Staphylococcus aureus* isolated from nasal swabs of buffaloes in Bangladesh; **Table S2**: Prevalence of antibiotic‐resistance genes of *Staphylococcus aureus* isolated from nasal swabs of buffaloes.

## Data Availability

The data that support the findings of this study are available from the corresponding author upon reasonable request.
