# Supplementary material for: Molecular Prevalence and Antimicrobial Resistance Patterns of Methicillin‐Resistant Staphylococcus aureus in Buffaloes from Bangladesh
Source: Vet Med Sci. 2026 Oct 16;12(6):e71295. doi: 10.1002/vms3.71295 (PMC13674740; doi:10.1002/vms3.71295)
Supplement: Supplementary file 1 [file VMS3-12-e71295-s001.docx]

**Table S1.** Antimicrobial classes, antibiotics, disc potencies, and zone diameter interpretative criteria (sensitive, intermediate, and resistant) used for antimicrobial susceptibility testing by disc diffusion method.

| **Antimicrobial class** | **Antibiotics** | **Disc potency (ug)** | **Interpretation** | | |
| --- | --- | --- | --- | --- | --- |
|  |  |  | **Sensitive (mm)** | **Intermediate**  **(mm)** | **Resistant**  **(mm)** |
| Penicillin | Ampicillin (AMP) | 10 | ≥29 | --- | ≤28 |
|  | Amoxicillin/  Clavulanic acid (AMC) | 20/10 | ≥29 | --- | ≥28 |
| Cephems including Cephalosporin | Cefoxitin (FOX) | 30 | ≥22 | --- | ≥21 |
|  | Ceftriaxone (CRO) | 30 | ≥23 | 20-22 | ≥19 |
| Aminoglycosides | Gentamicin (CN) | 10 | ≥15 | 13-14 | ≥12 |
|  | Amikacin (AK) | 30 | ≥20 | 17-19 | ≥16 |
|  | Streptomycin (STR) | 10 | ≥15 | 12-14 | ≥11 |
| Tetracyclines | Tetracycline (TET) | 30 | ≥19 | 15-18 | ≥14 |
| Macrolides | Azithromycin (AZM) | 15 | ≥18 | 14-17 | ≥13 |
| Quinolones | Ciprofloxacin (CIP) | 5 | ≥21 | 16-20 | ≥15 |
|  | Levofloxacin (LEV) | 5 | ≥19 | 16-18 | ≥15 |
| Folate pathway antagonists | Trimethoprim- sulfamethoxazole (SXT) | 1.25/23.75 | ≥16 | 11-15 | ≥10 |
| Phenicol | Chloramphenicol (C) | 30 | ≥18 | 13-17 | ≥12 |

**Table S2:** Prevalence of virulence genes of *Staphylococcus aureus* isolated from respiratory tract of buffaloes in Bangladesh

| **Virulence genes** | **Number samples tested** | **Positive samples** | **Prevalence** | **95% CI** |
| --- | --- | --- | --- | --- |
| Hemolysin A (*hla*) | 78 | 9 | 11.53% | 5.41, 20.78 |
| Staphylococcal enterotoxin A (*sea*) | 78 | 14 | 17.94% | 10.17, 28.28 |
| Intracellular adhesion-A (*ica*A) | 78 | 5 | 6.41% | 2.11, 14.33 |
| Fibronectin binding protein-A (*fnb*A) | 78 | 0 | 0.00% | 0 |

**Table S3:** Prevalence of antibiotic-resistance genes of *Staphylococcus aureus* isolated from respiratory tract of buffaloes.

| Genes | No. of isolates examined | Positive isolates | Percentage | 95% CI |
| --- | --- | --- | --- | --- |
| *aac(3)- IV* | 78 | 12 | 15.38% | 8.21, 25.33 |
| *tetA* | 78 | 7 | 8.97% | 3.68, 17.62 |
| *strA* | 78 | 4 | 5.12% | 1.41, 12.61 |
| *sul1* | 78 | 8 | 10.25% | 4.53, 19.21 |
